# Human resources: the Cinderella of health sector reform in Latin America

**DOI:** 10.1186/1478-4491-3-1

**Published:** 2005-01-19

**Authors:** Núria Homedes, Antonio Ugalde

**Affiliations:** 1 University of Texas – Houston, El Paso Regional Campus, Texas, USA; 2 Department of Sociology, University of Texas – Austin, Austin, Texas, USA

## Abstract

Human resources are the most important assets of any health system, and health workforce problems have for decades limited the efficiency and quality of Latin America health systems. World Bank-led reforms aimed at increasing equity, efficiency, quality of care and user satisfaction did not attempt to resolve the human resources problems that had been identified in multiple health sector assessments. However, the two most important reform policies – decentralization and privatization – have had a negative impact on the conditions of employment and prompted opposition from organized professionals and unions. In several countries of the region, the workforce became the most important obstacle to successful reform.

This article is based on fieldwork and a review of the literature. It discusses the reasons that led health workers to oppose reform; the institutional and legal constraints to implementing reform as originally designed; the mismatch between the types of personnel needed for reform and the availability of professionals; the deficiencies of the reform implementation process; and the regulatory weaknesses of the region.

The discussion presents workforce strategies that the reforms could have included to achieve the intended goals, and the need to take into account the values and political realities of the countries. The authors suggest that autochthonous solutions are more likely to succeed than solutions imported from the outside.

## Introduction

Health reforms that aim at increasing efficiency, quality and users' satisfaction need to take into consideration human resource issues, because the health sector is labor-intensive and the performance of health systems depends on qualified and motivated workers [1-4]. At the same time, the support of the workforce is crucial to ensure successful implementation of reforms.

In Latin America, the need to improve the performance of the workforce had been pointed out in many health sector assessments conducted in the 1970s and 1980s by the United States Agency for International Development (USAID), the World Bank (WB), other agencies and independent researchers. (See for Argentina [5-7], for Bolivia [8-10], for Brazil [11], for Chile [7,12], for Colombia [12-14], for Costa Rica [15], for the Dominican Republic [16-18], for Ecuador [19,20], for El Salvador [21], for Guatemala [22,23], for Mexico [12,24-26], for Nicaragua [27], for Panama [28,29], and for Uruguay [7].)

From these reports and studies, and notwithstanding the differences among the countries in the region, we can summarize the problems present during the 1970s and 1980s as follows:

• The skill mix of health personnel was often inadequate to meet the needs of the communities, and highly qualified staff often performed tasks that could be conducted by less-trained providers. The health systems of the region were characterized by an excess number of medical specialists and insufficient numbers of other professionals such as primary care providers, nurses, pharmacists, public health specialists, epidemiologists, health economists, accountants, social workers, administrators, communication experts, planners, health educators, nutritionists, physical therapists and sanitary engineers;

• There was an over-concentration of qualified health personnel in hospitals and urban centers, coupled with shortages in poor neighborhoods and rural areas;

• A large majority of physicians held at least two jobs, one in the government and one in the private sector. In countries with fragmented health systems, physicians could even have three jobs: they worked part time for the social security institute, they worked for the ministry of health, and also held a private practice. Dual or triple employment generated conflicts of interests; physicians used the public sector to draw patients for their private practice, and their productivity in the public post was low and absenteeism high;

• Human resources management systems were weak, largely due to dispersal of accountability: in many countries the terms and conditions of employment were under the control of the public service commission or the ministry of finance, and the education of human resources was under the control of the ministry of education or the private sector. Ministries of health did not have any input in determining the types and number of persons to be trained, and their involvement in hiring and managing the health workforce was limited. Health managers handled relations with the labor unions, had some limited supervisory roles, ensured organizational adherence to recruitment policies, and were responsible for some training.

• Salary increases were generally based on years of service. In the majority of countries, central labor unions negotiated working conditions directly with governments and signed collective agreements that left administrators with little room to compensate workers according to performance;

• Personnel decisions (hiring and promotion) were too often guided by favoritism, political dictates, and nepotism;

• Health professionals were insufficiently committed to the public system due to the conflict of interests mentioned above, poor personnel management systems and the perception that wages were low;

• The medical profession strongly dominated the definition of health sector policies and the regulation of the conditions of practice of all health professions;

• Communication between providers and patients was poor, and providers and service users had very different social and cultural backgrounds. In countries with Amerindian-speaking populations, providers did not speak their languages;

• The regulation of training institutions and conditions of practice was weak;

• The training of health promoters and other auxiliary personnel such as dental assistants, midwives, laboratory technicians, equipment maintenance and repair technicians, and pharmacy clerks was poor or non-existent, thus their performance was poor.

According to the literature reviewed, these conditions led to low productivity and efficiency; inadequate equipment; shortages of supplies and drugs; unmotivated and inadequately trained staff; questionable quality of care; and low users' satisfaction.

By the mid-1970s, the need to reform the human component of the health services was very urgent, and the urgency increased with the severe economic downturn that countries of the region suffered during in the early 1980s. The size of the Latin America health labor force (about nine million [30]) implied that reformers attempting to resolve the human resources problems mentioned above needed to dedicate a large amount of time and resources to it.

This paper reviews the impact of the health reforms carried out under the leadership of the World Bank. Data come from a review of the literature including the leading Latin American and non-Latin American journals, monographs, documents found in ministries and reform offices, technical reports, papers presented at conferences and fieldwork carried out by the authors between 1980 and the present in Bolivia, Colombia, Costa Rica, Dominican Republic, Ecuador, El Salvador, Honduras, Mexico and Peru.

## The World Bank's neoliberal health sector reforms

In the early 1980s, with a few exceptions, countries in the region entered a severe economic downturn. The International Monetary Fund and the World Bank required – as a condition for new lending and for refinancing the debts – reduction of public spending. The impact of structural adjustment programs on health services was severe and compromised the ability of governments to maintain the physical infrastructure of the facilities, provide the necessary supplies and equipment, and maintain competitive salaries for the workforce.

The World Bank took advantage of this situation to provide loans to the ministries of health and social security funds. Together with the loans, the Bank offered guidelines for the reorganization of the health services according to the ideological economic principles held by the institution.

The World Bank had started its activities in the health sector in the early 1970s, and by the early 1990s it was the world's largest health sector lender. In 1999, the total accumulated value of health, nutrition, and population loans worldwide amounted to USD 16.8 billion (in 1996 dollars) [31]. By the late 1980s, the Bank placed health financing at the center of its health policy dialogue with borrowers. In 1987 the Bank proposed four changes [32]: to impose user fees at government facilities; introduce insurance or other risk coverage systems; use nongovernmental resources more effectively; and decentralize planning, budgeting and purchasing for government health services [32] A few years later, it looked at the roles of the government and the market in the health sector, and described the main components that have guided most World Bank-led health reform efforts [33]. These principles were reiterated in 1997 [34].

The underlying principles of the Bank-led reforms include the belief that the private sector is more efficient than the public sector, and that decentralized administrative units are better-equipped to respond to the needs of the population than centralized governments. The Bank also proposes to limit government financing to a basic package of services, to be determined for each country by cost-effectiveness studies and the country's ability to pay. Services included in the basic package are made available at no cost to the indigent population; governments and the rest of the population subsidize them. In the Bank's health reform model, the role of the state is limited to that of a regulator of the health care market. The United States health system is closer to the World Bank model than the national health services or social security funds of other industrial nations.

The World Bank-led reforms did not include strategies to correct the human resources problems presented earlier, even if some of them had been identified by Bank-sponsored studies; the World Bank reformers believed that market forces would resolve them. As we will see, the contrary occurred and privatization and decentralization had some negative consequences for the workforce. The reliance on the market also hid the structural problems that needed to be taken into account at the time reformers designed human resource policies. The design and implementation process which were characterized by secrecy, generated dissatisfaction among health workers.

We have organized our analysis of human resources during the reforms under five categories: resistance of the workforce to the implementation of a market-oriented health care model; faulty implementation processes; inadequately trained personnel in managerial positions and in service delivery; institutional and legal dimensions including insufficient and faulty information, lack of financial resources, and civil service statutes; and weak regulatory legislation to ensure the quality of professionals and the performance of the sector.

## Resistance to the implementation of a market-oriented health care model

The neoliberal health reforms intended to change the values that had inspired the Latin American systems and the relations between the government and the health workforce. Thus, health would no longer be considered a right; only the insured would be entitled to receive a broad array of services. Health workers would lose the protection they had enjoyed as public servants and become part of a flexible workforce (Table 1).

**Table 1:** Resistance of the workforce to a market-oriented health care model

a. Health is not a right, and the reformed system is no longer based on the principles of solidarity and access to health care.
b. Health workers become part of a flexible workforce and are encouraged to compete, instead of collaborate, among themselves.
c. Worker unions lose power to influence the system and negotiate work conditions of behalf of affiliates.
d. The reform is an abdication of the responsibilities of the government to protect the population.
e. Physicians fear losing their professional autonomy.

Humans tend to resist change, but opposition to health reform was compounded by conflicts with the value system that inspired it. Most Latin American constitutions recognize health as a right and most governments had adopted Alma Ata's primary health care principles as their strategy to promote Health for All. Health policies were based on the principle of solidarity and fostered cooperation among health workers and also between them and other workers in related sectors such as education and agriculture [35]. It was assumed that health workers would do the best for their clients out of a sense of personal duty [4].

Human resource changes were needed to implement Alma Ata, but the budget reductions required by the World Bank caused a significant deterioration of working conditions. As their purchasing power worsened, health workers intensified undesirable behaviors to increase their income. These included levying illegal fees, diverting patients from the public sector to their private clinics [15], using public supplies and equipment for personal profit [36], and reducing their productivity in the public sector [15].

As indicated, Latin American physicians have supplemented their income from the public system by means of their private practice. Physicians value the stability and fringe benefits of a public post, but as the public delivery system deteriorated in many countries, private clients became their main source of revenue. By the late 1980s in most Latin American countries (with the exception of Argentina, Costa Rica, Cuba, Dominican Republic, Guatemala and Panama), more than half of the health expenditures, including the cost of medicines, occurred in the private sector [37], and in most countries of the region, personnel expenditures accounted for about 60% to 70% of the public health budget [38].

One of the objectives of the neoliberal reforms was to create a more flexible labor force by decreasing the number of tenured public employees and increasing the number of temporary personnel. This change threatened: the job security of the civil service, a very important dimension in countries with little political stability and where workers are frequently exposed to arbitrary political removals; the providers' income, which now would depend on their ability to compete for contracts and clients; and that which workers expected from their employer (recognition, opportunities for self-actualization and promotion). The promoters of health reforms failed to acknowledge that in a politically unstable region, civil service tenure was necessary to maintain an efficient, productive, and loyal labor force. Predictably, these threats triggered the opposition of professional associations and unions, led to strikes, and lowered productivity during the reform process.

Health workers of most countries of the region are unionized [39]. The unions protect the workers from the politicization of the appointments and promotions, and their leaders regularly engage in collective bargaining with the managers of the public sector. The stability provided by the civil service status facilitates the formation of strong union leadership: union leaders remain in office longer than policymakers.

Labor unions anticipated that the decentralization and privatization of the sector would have a negative impact on their membership and their bargaining power. Labor unions in Bolivia, Ecuador, El Salvador, Mexico, Nicaragua, Peru and Venezuela expressed their opposition to the reforms on the grounds that they were an excuse for governments to relinquish their constitutional responsibility to ensure access to care and would lead to dramatic changes in work conditions [39]. In some countries, such as El Salvador and Mexico, unionized workers successfully stalled or delayed the implementation of the reform.

Analysts of the health reform policies agree with the workers' concerns. Segall [4] asserts that a market system does not nurture the service ethic that should characterize the workforce and leads providers to adopt self-seeking behaviors instead of working in the interests of the patient or the community. Others worry that the uncertainties generated by the reform, the stress and demands added to the workforce and the misalignment between the values of the workers and those of the reformed system are very detrimental to the workers' motivation [1,40]. According to Rigoli and Dussault [41] the unions' fears were justified; union membership appears to have decreased in recent years.

In addition, health professionals strongly opposed the idea of having their professional autonomy limited by professional administrators who would force them to adhere to diagnostic and treatment protocols based on economic principles rather than on technical criteria. The cool reception that Mexican professional associations offered to foreign insurance companies and health maintenance organizations is a good example of this concern [42].

## Institutional and legal constraints

The large majority of countries in Latin America do not have accurate information on the numbers and distribution of the health workforce. There is no centralized entity responsible for gathering information on the health personnel practicing in the country or in the different geographical regions, and even when only public sector workers are examined, the numbers collected at different administrative levels differ (people may move to a different location, and regional or local governments hire additional staff without reporting to the central authorities). The reforms worsened the possibility of having accurate information. If the system is decentralized and the majority of providers are in the private sector, the sources of information from which human resources data will need to be collated multiplies. The challenge may be even greater if each agency gathers the information differently, and without this basic information it is very difficult to engage in human resources development planning (Table 2).

**Table 2:** Institutional and legal dimensions

a. Lack of accurate information on the availability of human resources and their distribution.
b. Civil service status limits the capacity of managers to change the working conditions of personnel.
c. Decentralizing human resources is expensive: homologation of salaries and benefits; hiring of additional personnel.
d. Decentralized governments have limited ability to manage personnel to respond to the needs of the population.

Civil service laws and negotiated agreements with worker unions have limited the freedom of public sector managers to reorganize the workforce; managers had to "administer the rules" [43] issued elsewhere. For instance, managers could not change the status of public sector employees to flexible contract workers, dismiss employees, increase workloads or change the working schedule. They may also have had difficulties rewarding their employees because the salaries and the conditions of employment were set outside the health sector by the public employment commission, the ministry of labor, or the ministry of finance or their corresponding decentralized entities. Reformers did not foresee these limitations.

Managers overcame these constraints by hiring additional personnel under flexible contracts. In Brazil there are now about 15 different types of contracts for public sector employees; a significant expansion of the use of temporary workers and contracts without fringe benefits has been reported in Argentina [44], Colombia [45], Ecuador [2], El Salvador [30], Panama and Peru [2]. Funds for the temporary positions have different origins, including extrabudgetary government allocations, revenues generated through user fees, or savings from positions left empty due to retirements or administrative leaves.

Transferring human resources to other politico-administrative levels was more difficult than anticipated. Kolehmainen-Aitken [46] and Homedes and Ugalde [47] have suggested that this is the most complex part of the decentralization process. The fear of transfers caused discontent and anxiety. On the other hand, managers did not want to absorb everybody who was transferred [46]. The decentralized personnel felt insecure about the new reporting mechanisms and the new managers' expectations, and vulnerable to political crossfire.

Decentralizing human resources is expensive. Prior to decentralization – in countries such as Bolivia, Colombia and Mexico – local or regional governments frequently hired additional personnel under different pay scales and benefit packages than those used by the central government. After decentralization, each decentralized administrative unit had to set pay scales, fringe benefits, performance assessments and reward systems for its workers. Because it was not possible to lower the salaries and reduce the benefits of workers with higher salaries and benefits – generally federal/national workers – it was necessary to bring the salaries and benefits of all the workers to the level of those who had the most generous package. Higher salaries and benefits represent an increase in the fixed costs of the system for an indefinite period.

Moreover, the creation and/or strengthening of the decentralized administrative structures are often not accompanied by a decrease in the number of federal employees, further raising the costs of personnel to the system [48]. In Mexico, from 1985 to 1987 the cost of transferring federal employees to 14 states was 140,000 million pesos (approximately 452 million US dollars) [49], and in Colombia the World Bank indicated that the cost of transferring state and departmental health personnel to Cali's Municipal Health Secretariat was prohibitive [50].

Decentralization has been promoted under the assumption that the proximity of decision makers to the communities facilitates providing services more in accordance with the needs of the community. But the decentralized structures' ability to respond to local needs has been constrained by: the civil servants they inherited, who are often inadequate in terms of skill mix and geographical distribution; the conditions of employment imposed by other government sectors and the unions; and the local elite, who do not have in mind the health needs of the communities and who lobby to have relatives and friends hired by local health authorities.

In several countries of the region, decentralization has broadened the urban-rural inequities in the distribution of personnel and in the quality of services [51-54]. Rich decentralized units are able to offer better working conditions and attract qualified personnel from poorer municipalities, a sort of brain drain. Similarly, the development of the private sector also tends to drain qualified resources from the public sector [43].

## Inadequately trained personnel

Countries considered the human resource implications of reform only when they faced resistance from the unions or realized they did not have the financial and human resources required to implement the reform [55]. The health reform plan in Costa Rica recognized the ambiguity of the policies and procedures related to human resources but did not modify them. As planned, incentives were established to increase workers' productivity and the use of short-term contracts. These two strategies ended up triggering greater grievances on the part of public sector employees [43].

The human resources units of the health ministries and the regional and local administrations were inadequately prepared for the reform (Table 3). Traditionally they had had a narrow scope of responsibilities, often limited to managing the relationship with the unionized workforce, ensuring compliance with national/provincial policies on recruitment and deployment of personnel, and organizing continuing education activities [56]. The personnel attached to these departments generally had limited or no human resources development training [30,57].

**Table 3:** Inadequately trained personnel

a. Human resources units are not adequately staffed, especially to manage change.
b. Lack of management experts, especially experts in insurance systems and contract managers.
c. Insufficient numbers of people trained in primary health care and public health related fields.
d. Training centers unable to produce personnel to operate the reformed health system.

The tasks required by the reform overstretched the capacity of these units. Included among these tasks were: the development of new organizational structures, defining new job descriptions, reassigning responsibilities and designing new reporting systems, establishing performance evaluation methods and assisting all decentralized units to carry out similar duties in their jurisdictions.

The decentralization of personnel often unveils rivalries and discontent among personnel who feel unfairly treated. All these issues need to be addressed, negotiated and resolved; and most human resources units were ill-prepared to lead the process [56]. For example, when the health system in Bolivia was decentralized, the salaries for several workers in Santa Cruz were delayed for months. Costa Rica introduced performance-based contracts between the Social Security Fund (Caja Costarricense de Seguro Social) and the health units, but according to a senior executive of the Ministry of Health, the targets were set at minimum levels and some units decreased their level of production [personal communication with the authors].

The mismatch between the abilities of the workforce and the needs of the system documented in the 1980s still prevails. The lack of coordination between training institutions and employers is at the root of the problem, along with weaknesses in the regulation of health professions and the dominance of physician's groups on the policy making process.

According to Bach [56], shortages of personnel trained in disciplines such as primary health care, health economics, public health, health communication, health education, nutrition, and environmental engineering continue to severely limit the possibilities for improving the quality and efficiency of the health care system. Only in very few instances has reform included the human resources development activities to address these issues. For example, Costa Rica trained multifunctional teams for their lowest-level facilities and promoted the training of general doctors instead of specialists [58]. The National Autonomous University of Mexico modified its curriculum to promote family medicine; it introduced public health concepts and interdisciplinary experiences around issues related to promoting the health and wellbeing of the elderly and protecting workers from occupational hazards [59].

In most countries, managerial positions were traditionally given to physicians with little or no management training [55]. The neoliberal reforms require managers and staff with experience in specific dominions such as insurance, capacity to write contracts and enforce them, ability to monitor performance, knowledge of performance-based reimbursement systems, and expertise in health services research to evaluate progress. Decentralization to the state and municipal levels generates the need for even more managers, and countries that have given autonomy to hospital executives to manage their human and financial resources face additional challenges. With the exception of Chile, countries in the region had limited expertise in private health insurance [60]; Costa Rica, the Dominican Republic, Mexico and Venezuela engaged expensive foreign technical assistance to develop performance-based contracts and management information systems.

The promoters of the reforms recognized the need for good managers but, because they had heavily criticized the public sector and questioned its role, it was difficult to recruit qualified staff into managerial positions and, in turn, it was difficult for those recruited to motivate and retain qualified staff [56,57]. Most health reform projects included management training.

The Pan American Health Organization [61] evaluated 15 such training programs and concluded that the training did not change the performance of the systems and that for only two projects was management capacity improved. The reasons for the failure included: difficulties in recruiting trainers; inability of the local universities to respond to the needs of the projects; inappropriate selection of training participants; conflict between project units and the ministries of health; political interference; and the absence of a human resource development plan. Theoretically the deficiencies in formal training can be corrected with supervision and continuing education activities, but this did not occur in Latin America. The authors of the report emphasize the need for countries to develop comprehensive human resources development plans to ensure the efficacy and sustainability of the training programs.

After having spent millions of dollars in training and infrastructure, the region's capacity to manage contracts is still limited [62], and when contracts are in place, they are expensive to administer and the legal system is insufficiently developed to enforce them. In Colombia, most public hospitals were unable to compete with the private sector and are now bankrupt [63]. Poor management in decentralized entities has been considered one of the main reasons for the decentralization failure [64,65].

## Faulty reform implementation process

Countries in the region used a top-down approach to define and implement reform. The implementation was often led by a handful of top health executives, newly created reform offices, the political elite and international agencies, which in turn contracted for the technical assistance of international consultants and universities closely aligned with the neoliberal ideology of the World Bank. In general, there was little interest in involving professional associations, unions or even local universities in this process [2,56] (Table 4).

**Table 4:** Faulty reform implementation process

a. Lack of involvement of professional associations and labor unions in the definition of the reform.
b. Secrecy surrounding definition of the reform raises suspicions among those responsible for implementing it and predisposes them to resist the changes.
c. Lack of transition plan.

In Costa Rica the labor unions were involved initially in the reform, but their influence was undermined by the World Bank and the Inter-American Development Bank, which adopted a more closed, centralized decision-making style [58]. According to a top executive of the Ministry of Health in El Salvador, the group preparing the reform operated in secret, and in his view, the secretive process was desirable because if health workers had participated they would have created obstacles to its implementation [66]. Indeed, it appears likely that the labor unions and professional associations in this country and many others would not have approved neoliberal reforms.

Some authors have offered a different interpretation of the exclusion of the workforce and assert that it was the complexity of the human resources issues and the need to involve many players (the ministries of education, labor, finance and health, and professional associations and unions) in their solutions that led reform promoters to ignore the labor force and other stakeholders [30].

Regardless of the underlying motivation, the secrecy that surrounded the process of defining and implementing the reforms produced rumors, confusion and hostility against the reform among civil servants and professional groups [30,39,67,68]. The objectives and processes by which reforms were introduced were never made clear; and often the reforms were perceived as responding more to ideological concerns of international organizations, in particular the International Monetary Fund and the World Bank, than to the needs, resources and sociopolitical realities of the countries [57]. The president of the Medical Association of El Salvador said that his association had tried to obtain information about the reform for over a year and that all his knowledge was based on "rumors and guesswork that led nowhere" [66].

In addition, health reformers did not consider the strategies and resources needed during the transition and early stages of implementation; such as allocating new financial resources and establishing clear communication channels. In addition, as discussed in the previous section, the reforms failed to adequately train managers who could lead the transition to a new management system [40].

## Inadequate regulatory system to ensure high-quality training and health providers

The need for regulation increases in health systems where the private sector plays a prominent role. In Latin America it was not until the early 1990s, largely as a consequence of the health reforms, that health policy makers became aware of the urgency to regulate the health system. A regulatory system includes adequate regulations, the institutions to enforce them, and a judicial system that ensures a timely response in the event of conflict. Enforcing regulations is important to guarantee that trained personnel provide safe and adequate services (Table 5).

**Table 5:** Inadequate regulatory framework to ensure the quality of professionals and the performance of the sector

a. Limited quality controls in training institutions.
b. Physician-dominated field that precludes other professional groups from being recognized as health care providers within the official health care system.
c. Limited accreditation of health care professionals.

Prior to the reforms, the regulatory systems were poorly developed and, when they existed, they were not tailored to the needs of consumers and enforcement was very limited [69]. For instance, the licensing of providers was a purely bureaucratic formality with no assessment of qualifications. The patronage and bossism observed in many countries were further expressions of regulatory deficiencies [70].

The number of medical schools has grown spectacularly in the last two decades and, in most countries, there were no mechanisms to ensure the quality of the training institutions or to test the abilities of the graduates. The number of medical schools in Chile grew by 68% between 1992 and 2000, in Peru the growth was also by 68%, in Argentina by 61% and in Brazil by 21%. The growth occurred mainly in the private sector [71]. Costa Rica had no private universities until the 1970s; now there are 70, several of which train health professionals [72].

By its very nature the regulation of the health profession relies heavily on the opinions of professionals and especially on physicians, who in turn place a great value on their autonomy and have had little interest in responding to social and political demands [55]. Medical associations have traditionally opposed health reforms and have had a very strong influence on health policy-making [29,66,73,74].

The dominance of physicians has alienated other professions such as therapists, nurses, pharmacists, optometrists and psychologists [75]. For example, in Chile the government proposed to train and use more optometric technicians, but medically trained ophthalmologists opposed the proposal. After a long negotiation process involving ophthalmologists, optometric technicians, insurance companies, universities, parliamentary representatives and consumers, an agreement was reached. The four-year trial period allows optometric technicians to expand their scope of practice while medical schools take in more ophthalmologists for training [58].

The regulation of the health professions has a long way to go in Latin America, and it is probably impossible to establish sustainable regulatory mechanisms in the absence of political and judicial reforms; for the system to work, it needs to free itself from political interference [70]. Some argue that the separation between professional associations and licensing bodies [76] must be increased, and there is general agreement that the perspective of the general public [75] must be included.

## Consequences of the health reform on human resources

Bach [56], Brito et al. [77], Dussault and Dubois [78], Rigoli and Dussault [41] have identified human resources issues as the main obstacle for the success of the reforms. The neoliberal health reforms did not solve the workforce problems that had previously been identified, and created additional ones that have had a negative impact on the health systems (see Table 6).

**Table 6:** Consequences of the health reform on human resources

a. Working conditions have worsened, and talented workers migrate to the private sector or to other countries.
b. The motivation of workers has deteriorated.
c. Productivity and quality may have deteriorated.
d. The uneven ratio of specialists to primary physicians has not changed.
e. The uneven distribution of personnel (hospital and urban bias) persists.
f. Corruption has not decreased.

The implementation of the reforms has been uneven in the Latin American region. Technical, logistical, political and financial problems have surfaced everywhere. While most countries decentralized, a few – such as Colombia and Chile – managed to significantly expand private insurance and, with the exception of Brazil, very few have engaged in large contracts with private sector providers. The most salient feature has been significant changes in hiring modalities. In Brazil there are 15 different types of contract arrangements [30] and in Peru the need to expand service coverage led to hiring 10 000 health professionals (physicians, nurses and technicians) between 1992 and 1996 under temporary contracts without social security; by the late 1990s about 12% of the health workers did not have social security [77]. Health workers in Ecuador have suffered wage reductions, in Mexico with decentralization the states have increasingly hired temporary workers [47], and in Argentina there has been a rise in precarious contracts, even fraudulent ones, such as full-time jobs under the label "autonomous professional" [30].

Another important result of the reform is the surge of multiple jobs, particularly in Argentina, Brazil, El Salvador, Panama, Peru, Uruguay, and to a lesser degree Chile [30]; that has caused stress and dissatisfaction among physicians.

A survey of nurses conducted in Argentina, Brazil, Colombia and Mexico [44] revealed that the reforms increased stressful conditions at work, job dissatisfaction, insecurity from flexible contracts, malpractice concerns, inter-institutional migration, and new bureaucratic tasks for which nurses were not trained. The nurses specifically mentioned that they needed to do more work in less time with fewer staff; and complained about excessive paperwork, including billing, and about having less time for direct patient care than before the reform. One of the nurses who participated in the study said "Patients may feel that we really don't care that much about them, because we just don't have enough time to spend with them and really know what is going on [44]." In a different survey, nurses who had gone through reform restructuring held more negative perceptions of patient care than those who had not; and they also expressed a higher desire to unionize [41].

Furthermore, according to the Tavistock Group [79] "cooperation throughout a health care system can produce better outcomes and much greater value for individuals and for society. Such cooperation requires agreement across disciplinary, professional and organizational lines about the fundamental ethical principles that should guide all decisions in a truly integrated system of health care delivery." If this statement is correct, by fragmenting the system through privatization and decentralization, and by introducing competition among health professionals, the neoliberal health reforms have compromised the quality of care.

Similarly, one of the basic principles of neoliberal reforms – that the efficiency of the system will increase by using flexible contracts and rewarding productivity – is not supported by the data. The World Bank conducted an evaluation of civil service reforms in 15 countries and concluded: "None of the cases reviewed so far have revealed any empirical evidence that the Civil Service Reform and related Technical Assistance Loans have succeeded in fostering the needed change in work attitudes, ethics and organizational culture that could lead to greater efficiency/productivity in the civil service" [1].

Research has also uncovered problems with using performance-based payment schemes. For example, in health it is often difficult to establish who is responsible for the outcome. Costa Rica implemented a pilot project in Barva de Heredia in which physicians received an incentive based on productivity, but this was not extended to the nurses and the other clinic staff [80]. The project increased the costs to the system without increasing the productivity of physicians or the quality of care, and as a result the government halted its plans to extend the model to other health facilities.

Health providers can also manipulate the information to maximize their benefits rather than the well-being of the patients; and substandard working conditions rather than workers' action may be responsible for a poor outcome [81]. Mexican providers opposed a malpractice evaluation system because they did not want to be held liable for errors due to equipment deficiencies and lack of supplies [42]. Health workers in Costa Rica feared that the focus on productivity compromises the commitment to patients [58] and discourages the provision of services that require extra time, such as health education [2,55] and mental health counseling.

Establishing a valid and reliable merit-pay system is extremely complicated; placing too much emphasis on material rewards may displace more intrinsic motivators such as the pleasure of doing good, or caring for the patient. Bennet and Franco [1] even suggest that loyalty to the organization may decrease as the worker becomes aware of more lucrative opportunities with other employers. This could have serious consequences. Attracted by NGOs and the higher salaries of private hospitals, the most talented public servants could leave the public sector. Others raised questions about the sustainability of these strategies. In Brazil, productivity-based payment systems resulted in increased productivity, but the increase was not sustained over time and created competition among workers who were expected to collaborate [82]. For an interesting discussion on incentive systems and motivation in a different context, see Le Grand [83].

There is a belief among neoliberal economists that private sector workers are more productive and less corrupt than public employees. Recent hospital studies confirm that because of fear of termination, absenteeism is less frequent among non-tenured physicians hired through short-term contracts than among civil servants, but short-term contracts have not increased commitment to the institution [84]. Corruption continues to be pervasive in both private and public hospitals, and productivity differences between the private and public hospitals have not been documented [85]. Costa Rica has attempted to reduce the waiting lists by contracting for the provision of services with private groups, under the condition that the recipient of the contract is someone not working for the clinic that makes the referral, a condition that is often violated [72].

Decentralization can also be seen as a transfer of financial responsibilities from central government to local authorities, which has the potential to affect wages and job stability [39] and increase inequity. Poor local authorities cannot compete with the conditions of employment offered by wealthier municipalities and have difficulties in attracting personnel. In a decentralized system it is more difficult to structure career ladders, especially for workers who choose to locate in rural areas [81]; and decentralization can also exacerbate forms of patronage and political domination. The experience in many countries has proven that it is more difficult to resist the politicization of decision-making when health managers interact with local political leaders without central controls [46,57,70].

In decentralized health systems, particular attention needs to be paid to establishing good coordination among those responsible for the vertical program at the central level, the decentralized administrative units, and the clinical staff. The absence of good coordination may result in health workers' reporting to two supervisors: the person responsible for the vertical program and the supervisor of the health facility or region, as is the case in Mexico [47].

In sum, in the majority of Latin American countries, the neoliberal reforms have not made the health delivery model more responsive to the needs of the community; have not increased the productivity of health workers; have had a negative impact on working conditions and staff motivation; appear to have further compromised the quality of care; and have had a limited impact on the capacity to regulate the health professions and training institutions.

## Discussion

More recent studies suggest that many of the old workforce problems remain unresolved [56,70,86,87]. Even the World Bank, which promoted the reforms, has finally recognized [88] that the neoliberal strategies are not having the desired impact. The Bank questions the performance of the private sector and highlights the need to find the institutional arrangements and policies that best respond to local conditions and resources.

Health reform provided a perfect opportunity to promote and encourage workforce improvement. Prerequisites for the progress of such processes are political will, effective relationships between the educational and service-providing institutions, and the open collaboration of professional groups. However, the reforms had the opposite effect. The neoliberal orientation challenged the use of conventional regulation strategies because, by encouraging professionals to seek their own interest instead of the interests of society as a whole, it questioned whether society and regulatory bodies could continue to trust and have faith in the criteria expressed by health professionals.

Human resources account for the lion's share of health budgets, and poor performance had been identified as the main constraint to efficiency, quality of services and user's satisfaction. The values that guided the neoliberal reform and the privatization and decentralization initiatives worsened the problems affecting the Latin American workforce and added new challenges. The reform implementation process was also responsible for the failures. The promoters of the neoliberal health reforms underestimated the importance of involving professional organizations and unions in the planning and implementation of the reform efforts, raising suspicion and resistance among organized health workers. In addition, the reforms were designed in secret and implemented using a top-down approach. The only significant advance in human resource development in the last 10 years is the increased interest in strengthening the capacity to regulate training institutions and practitioners.

Solving the problems that affect human resources is no easy task, and probably the solutions are different for each country; ignoring the problems and hoping that the market will resolve them is a recipe for failure. Managing change is very complex; embarking on reform without having secured the collaboration of the workforce and ensuring the availability of sufficient qualified staff is irresponsible.

The exclusion of the organized labor force from reform discussions is indefensible; the collaboration and motivation of the health workers is essential to the reform, and the leaders of the organized groups can assist in informing and aligning the workforce [40,89].

Policy changes requiring a different skill mix of health personnel require careful planning because there is a significant time lag between deciding that there is a need to train additional professionals and having them available. For example, a 10% rise in the number of students in a medical school produces only a 2% increase in the supply of doctors 10 years later [78]. Most countries did include a training component, but as mentioned earlier it was insufficient, and part of the problem was that the reform implementation was rushed, without the benefit of field-testing the underlying theories, gathering evidence on the appropriateness of the strategies, or learning from the reform experience.

One thing that reforms could have done was to support interventions that, independently from the reform, some countries had designed to overcome workforce weaknesses. Some interventions were national programs, others were pilot projects, and there were also experimental projects. The following are examples of these autochthonous interventions.

To solve the urban-rural gap and improve equity, most ministries of health in the region created the obligatory rural health service known as pasantía or servicio social that requires physicians to spend one year in a rural health center prior to graduation or immediately after receiving their degree [35].

A few social security institutes have also found ways to reduce health inequities; such is the case of the Mexican and Ecuadorian Institutes of Social Security (IMSS and IESS). IMSS organized COPLAMAR, an extensive primary and secondary care program for poor rural populations, which brought general practitioners and specialists to rural areas. The Ecuadorian program known as Seguro Social Campesino offered primary care services for rural dwellers and, when needed, hospitalization at the Ecuadorian Social Security Institute; this program aimed at reducing the rural-urban gap in a country of which 70% of the population then lived in the rural areas [90].

Mexico created a training program for traditional midwives who worked in dispersed rural populations; the objective of the program was to enhance the quality of their services and reduce maternal and infant mortality [91]. The Ministry of Health of the Dominican Republic, in an effort to increase health equity, trained and deployed more than 5 000 health promoters in rural centers. The promoters periodically visited every rural household to monitor infant growth, promote nutrition and sanitation, and assist in immunization campaigns [35].

Costa Rica attracted the world's attention with the Open Walls Hospital, a program that required the specialists of a regional hospital to schedule – when needed – weekly visits to dispersed rural populations. The program also intended to convey the message to specialists that they were not different from other health workers and had an obligation to serve poor rural dwellers even when doing so would involve personal inconvenience [80].

To enhance the professional status of primary care practitioners, reduce referrals, and improve quality of care, IMSS created many positions in the specialty of family physician, forcing the medical profession to recognize the status of the new specialty. Colombia's Ministry of Health sent nurses to a graduate health education program taking advantage of a US fellowship program in order to diversify the human resource composition of the Ministry.

Some of these projects were successful; this was the case of family physicians and COPLAMAR in Mexico. However, the first 14 Mexican states that decentralized in the 1980s dismantled the program, transferring it to the states' departments of health to create the state health system, and the quality of rural health deteriorated rapidly [92].

Others function poorly, as is the case of the compulsory year of social service in all the countries where it was established. Similarly, the health promoters program in the Dominican Republic suffered from insufficient training, lack of continuous education, absence of efficient supervision, and poor remuneration, comments that can be extended to all health promoter programs of the region. The Seguro Social Campesino has suffered from inadequate financing, and plans to extend it to the entire rural population have been placed on permanent hold.

Other programs were discontinued because of indifference and lack of support from policy makers. Thus, the Hospital without Walls ceased after all Ministry of Health hospitals were transferred to the Social Security Institute. Due to budgetary problems, the Colombian Ministry of Health did not employ the health educators on their return from graduate school.

The health reforms would have provided a perfect opportunity to support and strengthen many of these and other autochthonous interventions. A proper course of action would have been to evaluate the projects that had been developed locally, identify their strengths and weaknesses, and establish their viability and sustainability. Through trial and error and with appropriate resources and incentives, many of them are likely to be more effective and less costly than foreign programs invented by those who hardly know the realities of developing countries and are inspired by ideological principles and questionable economic theories.

There is much more that needs to be done to improve the training and management of human resources for health, and very often the solutions depend on the collaboration of a wide range of stakeholders such as those who produce health workers, those who employ them, those who pay for their services, those who negotiate working conditions and those who define the standards of professional practice. It is no easy task and can be successfully accomplished only if there is strong political will, if there is openness and trust among all stakeholders, and if sufficient resources and time are allocated to this effort. Most countries of the region have the capacity to find appropriate solutions to the problems they are facing. Dussault [55] argued that change is possible only on the basis of shared values, and as we have seen, the values that inspired the neoliberal reform did not coincide with those expressed in the Latin American Constitutions and in the primary health care principles that had guided the development of the health sector during the 1970s and 1980s.

As Segall mentioned [4], it is important to recover the spirit of cooperation among health providers, and there is a need to take explicit steps to raise their motivation and patient-centered behavior. Without a motivated workforce, all other efforts to change the system may be even counterproductive. Policy makers and administrators will have to explicitly identify strategies that foster collaboration, inner motivation and work ethics, and this may require abandoning the market orientation of the neoliberal reforms and embracing the values that inspired the primary health care movement.

## Competing interests

The author(s) declare that they have no competing interests.

## Authors' contributions

Both authors have contributed equally to the design, data collection, data analysis, drafting and completion of this article.

## References

[B1] Bennet S, Franco LM (1999). Public sector health worker motivation and health sector reform: A conceptual framework.

[B2] International Labour Organization (ILO) (1998). Terms of employment and working conditions in health sector reforms.

[B3] Martineau T, Buchan J (2000). Human resources and the success of health sector reform. Human Resources for Health Development Journal (HRDJ).

[B4] Segall M (2000). From cooperation to competition in national health systems – and Back? Impact on professional ethics and quality of care. International Journal of Health Planning and Management.

[B5] World Bank (1987). Argentina: population, health and nutrition sector review Report n° 655-AR Washington, DC.

[B6] Leofanti FL, Chiesa ME (1988). Neuquén, Argentina: Provincial health policies and their results. Rural Health.

[B7] Scarpaci J (1994). The role of physicians in shaping national health insurance. Studies in Third World Societies.

[B8] USAID (1975). Bolivia health sector assessment La Paz.

[B9] Bastien JW (1987). Cross-cultural communication between doctors and peasants in Bolivia. Social Science and Medicine.

[B10] Bastien JW (1990). Community health workers in Bolivia. Adapting to traditional roles in the Andean Community. Social Science and Medicine.

[B11] McGreevey W Brazilian health care financing and health policy: An international perspective.

[B12] Hale FA (1988). The need to train physicians for rural primary health care in Latin America: Some family medicine experiences. Rural Health.

[B13] USAID (1972). Un análisis del sector colombiano de salud pública Bogotá.

[B14] Ugalde A (1979). The role of the medical profession in public health policy making: the case of Colombia. Social Science and Medicine.

[B15] Homedes N, Sanguinetty J, Rochwerger D (1988). Los recursos humanos del sector salud en Costa Rica.

[B16] LeBow R, McCarthy D, Cross P (1983). Project evaluation summary specific to the rural health delivery system (SBS) in the Dominican Republic Health Sector Loans I and II Evaluation and Recommendations.

[B17] Gómez Ulloa M (1985). El sistema de salud de la República Dominicana Unpublished report prepared for the Pan American Health Organization.

[B18] Ugalde A, Homedes N (1994). Physicians and underutilization of rural primary health services: the case of The Dominican Republic. Studies in Third World Societies.

[B19] Salazar González J (1981). Cuchucun: Soledad y sombra.

[B20] Lewis M, Sulvetta MB, La Forgia GM (1990). Measuring costs, efficiency, and quality in public hospitals: A Dominican Case Paper presented at the Second World Congress of Health Economics Zurich, September 10–14, 1990.

[B21] USAID (1978). Health sector assessment: El Salvador San Salvador.

[B22] USAID (1977). Health sector assessment: Guatemala Washington, DC.

[B23] Colburn FD (1981). Guatemala's rural health paraprofesionals Rural Development Committee Center for Internacional Studies Special Series on Paraprofessionals no 2.

[B24] Stebbins KR (1984). Second-class Mexicans: State penetration and its impact on health status and health services in a Highland Chinantec municipio in Oaxaca.

[B25] Flores Arechiga AF, Heras HR, Cantu IQ (1985). Family medicine: A medical care alternative for Latin America. Social Science and Medicine.

[B26] USAID (1981). Integrated rural health development Quito.

[B27] USAID (1976). Health sector assessment for Nicaragua Managua.

[B28] Ministerio de Salud (Panama) (1978). Atención primaria en salud La experiencia panameña.

[B29] La Forgia GM (1994). From domination to accommodation to confrontation: physicians and the state in Panama. Studies in Third World Societies.

[B30] Brito P (2000). Impacto de las reformas del sector salud sobre los recursos humanos y la gestión laboral. Pan American Journal of Public Health.

[B31] Govindaraj R, Reich MR, Cohen JC (2000). World Bank pharmaceuticals discussion paper.

[B32] World Bank (1987). Finacing health services in developing countries: An agenda for reform Washington, DC.

[B33] World Bank (1993). World Development Report 1993 Investing in Health.

[B34] World Bank (1997). Health, nutrition and population strategy paper Washington, DC.

[B35] WHO Informe del Director de la Oficina Regional de la OMS para las Américas/Oficina Sanitaria Panamericana ICPHC/AL/783 Alma Ata.

[B36] Ugalde A, Homedes N (1988). Towards a Rural Health Corps Concept: Lessons from the Dominican Republic. Journal of Rural Health.

[B37] Pan American Health Organization Health expenditure database for Latin America and the Caribbean.

[B38] Alwan A, Hornby P (2002). The implications of health sector reform for human resources development. Bulletin of WHO.

[B39] Maceira D, Murillo V (2001). Social sector reform in Latin America IDB, Research Department, Working paper 456.

[B40] Franco LM, Bennett S, Kanfer R (2002). Health reform and public sector health worker motivation: a conceptual framework. Social Science and Medicine.

[B41] Rigoli F, Dussault G (2003). The interface between health sector reform and human resources in health. Human Resources for Health.

[B42] Abrantes Pego R (2004). La lenta y difícil institucionalización de la reforma del sector salud en Sonora: 1982 a 2000.

[B43] Biscoe G (2001). Human Resources: the political and policy context Prepared for the global Workforce Strategy Group.

[B44] Guevara EB, Mendias EL (2002). A comparative analysis of the changes in nursing practices related to health sector reform in five countries of the Americas. Revista Panamericana de Salud Pública.

[B45] Schlette S (1998). Public service reforms and their impact on health sector personnel in Colombia.

[B46] Kolehmainen-Aitken RL (1998). Decentralization and human resources: implications and impact. Human Resources for Health Development Journal.

[B47] Homedes N, Ugalde A (2004). La descentralización de los servicios de salud en Nuevo León y Tamaulipas.

[B48] Olvera Santana L (2002). Análisis de la implementación de la descentralización de los servicios de salud en el Estado de Baja California Sur 1996–2000.

[B49] Kumate Rodríguez J (1986). La descentralización de los servicios de salud In La descentralización de los servicios de salud: el caso de México.

[B50] World Bank (1994). Colombia Toward increased efficiency and equity in the health sector Can Decentralization Help? Sector Report n° 11933-CO Washington, DC.

[B51] Holley J (1995). Estudio de descentralización de la gestión de los servicios de salud Territorio de Capinota, Bolivia Latin American Health and Nutrition Sustainability Project.

[B52] Gonzáles-Block MA, Leyva R, Zapata O, Loewe R, Alagón J (1989). Health services decentralization in Mexico: Formulation, implementation, and results of policy. Health Policy and Planning.

[B53] Duarte Quapper D, Zuleta Reyes MS (1999). La situación de salud primaria en Chile.

[B54] Larrañaga O (1997). Eficiencia y equidad en el sistema de salud chileno Serie Financiamiento del Desarrollo Proyecto CEPAL/GTZ Reformas Financieras al Sector Salud en América Latina y el Caribe.

[B55] Dussault G (1999). Human Resources Development: The Challenge of Health Sector Reform.

[B56] Bach S Human resources and new approaches to public sector management Presented at the meeting organized by WHO: Global Health Workforce Strategy, Annecy, France December 9–12, 2000.

[B57] Green A, Collins C (2003). Health systems in developing countries: public sector managers and the management of contradictions and change. International Journal of Health Planning and Management.

[B58] Egger D, Lipson D, Adams O (2000). Issues in health services delivery: Human Resources for Health WHO/EIP/OSD/002.

[B59] PAHO Human resources: a critical factor in health sector reform Regional Meeting San José, Costa Rica December 3–5, 1997.

[B60] Adams O, Hicks V Pay and non-pay incentives, performance and motivation Paper presented at the WHO meeting Global Health Workforce Strategy Group Annecy, France, December 9–12, 2000.

[B61] OPS (2001). Situación de los componentes educacionales en proyectos relacionados con los procesos de reforma del sector salud Programa de desarrollo de los recursos humanos Washington, DC.

[B62] Anonymous (2000). A Regulacao dos recursos humans o em saúde e a reforma do sector saúde em países da América Latina.

[B63] Tono T (2004). La crisis de los hospitales de Colombia Paper presented at the Conference: The Innovations in Health Financing, April 20–21, Mexico City.

[B64] La Forgia GM, Homedes N (1992). Decentralization of health services in Colombia A review of progress and problems A Report to the World Bank Washington, DC.

[B65] Ugalde A, Homedes N (2002). La descentralización de los servicios de salud en América Latina. Gaceta Sanitaria.

[B66] Homedes N, Paz C, Solas O, Selva-Sutter E, Ugalde A, Lloyd-Sherlock P (2000). Health reform in El Salvador: theory and practice. Health Care Reform and Poverty in Latin America.

[B67] Salinas H, Lenz R (1999). Las no reformas de salud en Latinoamérica Razones que explican su fracaso.

[B68] PSI (Public Service International) The IDB, the World Bank, labor rights and health care privatization in the Americas Washington, DC.

[B69] Bolis M (2002). Legislación y equidad en salud. Pan American Journal of Public Health.

[B70] Cunill N (1999). Mercantilización y neo-clientelismo o reconstrucción de la administración pública. Retos de las reformas de segunda generación. Revista Nueva Sociedad.

[B71] Milen A (2001). Análisis de los recursos humanos en los procesos de reforma.

[B72] Barahona R, Molina A, Rosales CE, Ministerio de Salud/ CCSS/OPS Observatorio de Recursos Humanos en Salud en Costa Rica: Avances y Perspectivas.

[B73] Ugalde A (1980). Physician's control of the health sector: professional values and economic interests. Social Science and Medicine.

[B74] Donahue JM (1986). The profession and the people: primary health care in Nicaragua. Human Organization.

[B75] Nicolau Girardi S (2000). Los dilemas de la reforma de la regulación del trabajo y de las profesiones de salud en la reforma del estado. Cuadernos Médico Sociales.

[B76] World Health Organization (2000). Strategies for assisting health workers to modify and improve skills: developing quality health care – a process change WHO/EIP/OSD/001 Geneva.

[B77] Brito P, Galin P, Novick M Labour relations, employment conditions and participation in the health sector Workshop on Global Health Workforce Strategy Annecy, France.

[B78] Dussault G, Dubois C (2003). Human resources for health policies: a critical component in health policies. Human resources for health.

[B79] Smith R, Howard H, Berwick D (1999). Shared ethical principles for everybody in health care: a working draft from the Tavistock Group. British Medical Journal.

[B80] Ugalde A, Homedes N (1988). Estudio de la consulta externa en Costa Rica.

[B81] Buchan J, Thompson M, O'May F (2000). Health workforce incentive and remuneration- a research review WHO/EIP/OSD/0014 Geneva: WHO.

[B82] Leal Cherchiglia M, Nicolau Girardi S, de Castro Vieira R, Bibiani de Aguilar Marques R, Mendes Werneck da Rocha P, Cimino Pereira LA (1998). Remuneración y productividad: el caso de la Fundación Hospitalaria del Estado de minas de Gerais, Brasil, 1992–1995. Revista Panamericana de Salud Publica.

[B83] LeGrand J (2003). Motivation, Agency and Public Health.

[B84] Di Tella R, Savendoff WD, eds (2001). Diagnosis corruption Fraud in Latin America's public hospitals.

[B85] Alcázar L, Andrade R, Di Tella R, Savendoff WD (2001). Induced demand and absenteeism in Peruvian hospitals. Diagnosis corruption Fraud in Latin America's public hospitals.

[B86] Gil Polonio C, Arbaje M (1996). Diagnóstico de la secretaria de estado de salud pública y asistencia social.

[B87] Ansal (Análisis del sector salud de El Salvador) (1994). Health sector reform in El Salvador: Towards equity and efficiency.

[B88] World Bank (2004). World Development Report 2004 Making Services Work for the Poor.

[B89] Reich MR (1995). The politics of health sector reform in developing countries: three cases of pharmaceutical policy. Health Policy.

[B90] Córdova Jiménez C El seguro social campesino en el Ecuador Paper presented at the I Jornada Internacional del Intercambio en Seguridad Social, Quito May 21–24, 1980.

[B91] Gallástegui B, Navarrete EL, Vera Bolanos MG (1994). Las parteras capacitadas por el IMSS en el Estado de México: el caso de Los Reyes La Paz. Población y Sociedad.

[B92] Birn A (1999). Federalist flirtations: the politics and execution of health services decentralization for the uninsured populations in México, 1985–1995. Journal of Public Health Policy.

